# Indications for Hospitalization in Children with SARS-CoV-2 Infection during the Omicron Wave in New York City

**DOI:** 10.3390/children9071043

**Published:** 2022-07-14

**Authors:** Karen P. Acker, Deborah A. Levine, Mathew Varghese, Katherine A. Nash, Arindam RoyChoudhury, Erika L. Abramson, Zachary M. Grinspan, Will Simmons, Alan Wu, Jin-Young Han

**Affiliations:** 1 Department of Pediatrics, Weill Cornell Medicine, New York, NY 10021, USA; kpa9002@med.cornell.edu (K.P.A.); del9096@med.cornell.edu (D.A.L.); mav9205@nyp.org (M.V.); err9009@med.cornell.edu (E.L.A.); zag9005@med.cornell.edu (Z.M.G.); 2 Department of Emergency Medicine, Weill Cornell Medicine, New York, NY 10021, USA; 3 Department of Pediatrics, Columbia University Vagelos College of Physicians and Surgeons, New York, NY 10032, USA; kan2123@cumc.columbia.edu; 4 Department of Population Health Sciences, Weill Cornell Medicine, New York, NY 10021, USA; arr2014@med.cornell.edu (A.R.); wis4002@med.cornell.edu (W.S.); alw4001@med.cornell.edu (A.W.)

**Keywords:** COVID-19, children, hospitalization, Omicron

## Abstract

The emergence of the Omicron variant was accompanied by an acute increase in COVID-19 cases and hospitalizations in New York City. An increased incidence of COVID-19-associated croup in children during the Omicron wave has been recognized, suggesting that there may be other changes in clinical symptoms and severity. To better understand clinical outcomes and health care utilization in children infected with SARS-CoV-2 during the Omicron wave, we performed a cross-sectional study in pediatric patients aged ≤18 years who were tested for SARS-CoV-2 in pediatric emergency departments within a large medical system in New York City from 2 December 2021 to 23 January 2022. We described the clinical characteristics and outcomes of pediatric patients who presented to the pediatric emergency department and were hospitalized with SARS-CoV-2 infection during the Omicron wave in New York City. There were 2515 children tested in the ED for SARS-CoV-2 of whom 794 (31.6%) tested positive. Fifty-eight children were hospitalized for a COVID-19-related indication, representing 7.3% of all COVID-19-positive children and 72% of hospitalized COVID-19-positive children. Most (64%) children hospitalized for a COVID-19-related indication were less than 5 years old. Indications for hospitalization included respiratory symptoms, clinical monitoring of patients with comorbid conditions, and exacerbations of underlying disease. Eleven (19%) hospitalized children were admitted to the ICU and six (10%) required mechanical ventilation. Children infected with COVID-19 during the Omicron wave, particularly those less than 5 years old, were at risk for hospitalization. A majority of hospitalizations were directly related to COVID-19 infection although clinical indications varied with less than a half being admitted for respiratory diseases including croup. Our findings underscore the need for an effective COVID-19 vaccine in those less than 5 years old, continued monitoring for changes in clinical outcomes and health care utilization in children as more SARS-CoV-2 variants emerge, and understanding that children are often admitted for non-respiratory diseases with COVID-19.

## 1. Introduction

The Omicron (B.1.1.529) variant of SARS-CoV-2 was first reported in New York City (NYC) on 2 December 2021. By 25 December more than 90% of NYC SARS-CoV-2 infections were due to Omicron [1]. In early reports from South Africa and Europe, Omicron was associated with rapid transmission [2] but less severe disease [3,4,5]. Disease severity also appeared to be lower in the U.S. [6]; however, the rates of COVID-19-associated hospitalizations in children and adolescents increased compared to prior waves [6,7,8]. Young children were particularly impacted with Centers for Diseases Control and Prevention surveillance data demonstrating that children 0–4 years had the highest hospitalization rates within the pediatric age group [7,9]. In contrast to earlier reports from South Africa that suggested Omicron was associated with a high rate of incidental infection [10,11], a majority of children and adolescents had COVID-19 as the primary reason for admission [7]. However, clinical diseases that drove these pediatric admissions have not been described. It is important to understand clinical reasoning behind pediatric hospitalizations with Omicron as it is now clear that Omicron led to at least one change in clinical presentations in children with a number of investigators reporting a rise in incidence of croup [12,13,14,15]. Croup is diagnosed clinically with a barking cough, stridor, and hoarseness and is caused by virus-induced subglottic airway inflammation or laryngotracheobronchitis. In this study, we evaluated clinical characteristics and outcomes for children with SARS-CoV-2 infection who presented to a pediatric emergency department (ED) within a multicenter healthcare system during the Omicron wave in NYC. We described the indications for hospitalization in children with SARS-CoV-2 infections and assessed the impact of vaccination on hospitalization.

## 2. Materials and Methods

### 2.1. Subjects and Setting

We conducted a cross-sectional study of patients aged ≤18 years who presented to a pediatric ED within a multicenter healthcare system in NYC between 2 December 2021 and 23 January 2022 and were tested for SARS-CoV-2. All patients aged ≤18 years who were tested for SARS-CoV-2 were included in the study. Patients were tested for SARS-CoV-2 upon presentation to the ED based on COVID-19 symptoms or exposure history. Additionally, all hospitalized patients were tested for SARS-CoV-2 per hospital policy. All SARS-CoV-2 testing was performed by real-time reverse transcriptase polymerase chain reaction (RT-PCR). Data were extracted from the COVID-19 Institutional Data Repository (IDR), which aggregates electronic health record data on patients tested for SARS-CoV-2 [16]. We defined the Omicron period as starting on 2 December 2021 to correspond with the first reported case in New York City.

This study was determined to be exempt by the Weill Cornell Institutional Review Board.

### 2.2. Variables

We extracted and evaluated patient demographic and clinical characteristics, including age, sex, race, ethnicity, vaccination status, and medical complexity as defined by the Pediatric Medical Complexity Algorithm [17]. COVID-19 vaccination status in the electronic health record is pulled from the New York Citywide Immunization Registry and from manual entry for those who were vaccinated outside the New York State. We ascertained clinical outcomes including disposition, need for respiratory support, primary diagnosis, prevalence of viral co-infection, and COVID-19 treatment. By manual chart review, we categorized children who tested positive for SARS-CoV-2 by RT-PCR and were hospitalized as either COVID-19-related or incidental infection (unrelated to hospital admission) using a previously described approach [18]. Infections were considered COVID-19-related if a patient had COVID-19 symptoms (e.g., respiratory symptoms, fever, and dehydration) and was admitted for symptom management. All admissions for respiratory compromise were considered COVID-19-related even in the presence of a viral co-infection as it was typically unclear whether respiratory symptoms were driven by COVID-19 or the co-infecting respiratory virus. Additionally, hospitalizations were considered COVID-19-related in patients with chronic medical conditions in the two following settings: (1) admission for clinical monitoring due to concern for progression to severe disease and (2) admission for an exacerbation of their chronic medical condition thought to be related to COVID-19 infection and with no clear alternative diagnosis.

### 2.3. Statistical Analysis

We reported demographic and clinical characteristics with descriptive statistics. Demographic and clinical characteristics were compared between hospitalized and non-hospitalized patients using Wilcoxon rank sum tests, Pearson’s Chi-squared tests, and Fisher’s exact tests. SARS-CoV-2 testing positivity and hospitalization rates between age groups were compared using Fisher’s exact tests. Rates of COVID-19-related hospitalization between unvaccinated and vaccinated children were compared using a risk ratio and 95% Wald confidence interval.

## 3. Results

**Figure 1:**
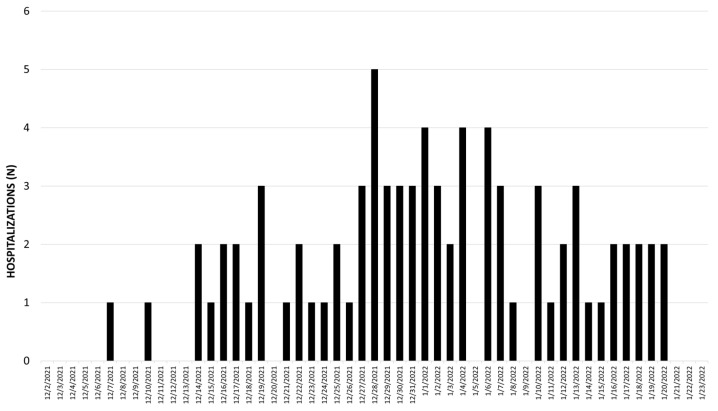
Number of COVID-19 positive hospitalizations per day from 2 December 2021 to 23 January 2022. Includes all children who tested positive for SARS-CoV-2 by RT-PCR and were hospitalized.

In our cohort, 2515 ED patients were tested for SARS-CoV-2, of which 794 (31.6%) tested positive (Table 1). Positivity rate was similar in all age groups (0–4 year 30%, 5–11 year 33%, 12–15 year 31.3%, and 16–18 year 36.3%, *p* = 0.19). Eighty-one (10.2%) patients who tested positive were hospitalized. Demographic characteristics, including median age, sex, race, and ethnicity, were similar between hospitalized and non-hospitalized COVID-19-positive patients. Hospitalized patients were more medically complex (complex chronic disease 68% vs. 24%, *p* < 0.001) than non-hospitalized patients. The first hospitalization in our cohort occurred on 7 December 2021 and hospitalizations peaked during the week of 28 December, followed by a gradual decrease (Figure 1).

Among 81 hospitalized children, 58 (72%) were hospitalized for a COVID-19-related indication and 23 (28%) incidentally tested positive. The rate of hospitalization for a COVID-19-related indication in SARS-CoV-2-positive patients was higher in the 0–4 y age group (9.5%) compared to children older than 5 years (5.2%, *p* = 0.02). Of the 58 admissions with a COVID-19-related indication, 28 (48%) patients had respiratory conditions, including 7 with croup, 5 with asthma exacerbations, 6 with pneumonia, 9 with exacerbations of underlying respiratory or cardiac diseases (e.g., chronic lung disease or congenital cardiac disease), and 1 with bronchiolitis (Table 2). Most patients with respiratory symptoms were less than 5 years old (22/28, 79%), including six of the seven croup cases. Nine (16%) patients with underlying chronic conditions and fever or mild respiratory symptoms were admitted for clinical monitoring due to concern for risk of progression to severe COVID-19 disease, and nine (16%) patients were admitted with exacerbations of underlying conditions that were attributed to COVID-19 infection (e.g., elevated creatinine in a child with end stage renal disease with no other explanation, vaso-occlussive crisis in a child with sickle cell disease, and anemia in a child with hereditary spherocytosis). The remaining eight (14%) children categorized as “Other” were previously healthy children admitted for fever and dehydration (7) or monitoring of chest pain (1). Four (7%) experienced neurological complications (bulging fontanelles (2) and increased seizure activity in epilepsy patients (2)).

Among the 58 children hospitalized for a COVID-19 related indication, 22 (38%) received respiratory support, of whom 6 (10%) required invasive mechanical ventilation. There was one patient death related to a chronic medical condition. Remdesivir was administered to 19 (33%) and steroids for COVID-19 treatment to 12 (21%) patients. Eleven children (19%) required treatment in an intensive care setting. Nine of these eleven children had underlying chronic conditions including congenital cardiac disease, chronic respiratory disease, and seizure disorders.

The majority (60%) of hospitalized patients were 0–4 years old and, therefore, ineligible for COVID-19 vaccination. In vaccine-eligible patients, there was no statistically significant difference in hospitalization rates between vaccinated versus unvaccinated patients (Table 3). Overall vaccination rate was low, and only 306 (25.6%) presenting to the ED for testing had received two or more doses of a COVID-19 vaccine. In hospitalized patients, only six (19%) were fully vaccinated, two of whom were admitted for COVID-19-related indications. One was immunosuppressed after a renal transplant and the other had sickle cell disease and concern for acute chest syndrome; neither required respiratory support or ICU admission. The other four vaccinated children were admitted with incidental COVID-19 infections.

## 4. Discussion

Between 2 December 2021 and 23 January 2022, corresponding to the NYC Omicron surge, more than 30% of pediatric patients presented to the ED tested positive for SARS-CoV-2. There was a proportional rise in hospitalizations that peaked during the week of 28 December in our cohort, which mirrors surveillance data from the NYC Department of Health and Mental Hygiene [19]. Of the children who were hospitalized, 72% were hospitalized for a COVID-19-related indication as opposed to an incidental infection. Overall, respiratory illness was the most common indication for admission. Recent studies have reported an increased rate of croup during the Omicron wave [12,13,14,15]. We did observe croup in 12% of hospitalizations yet the majority of hospitalizations were driven by other indications. Consistent with recent reports [7,9], children less than 5 years had the highest rates of hospitalization for a COVID-19 related indication, most commonly due to a respiratory syndrome, underscoring the urgent need for an effective vaccine in this age group. Children with chronic comorbidities were also highly impacted by hospitalizations due to need for clinical monitoring or exacerbations of underlying disease.

Differentiating patients who are hospitalized for COVID-19 indications from those with incidental infections is critical to understanding the epidemiology of COVID-19 waves. The combination of universal testing for hospitalized patients and high rates of asymptomatic or mild infections in children could lead to high rates of incidental infections that inflate the reporting of COVID-19 hospitalizations. In our cohort, 72% of COVID-19-positive hospitalized children were admitted for a COVID-19-related indication, which included management of symptomatic disease including respiratory syndromes, clinical monitoring for progression to severe COVID-19 disease, and exacerbation of underlying chronic diseases. This reflects a similar rate reported by the Coronavirus Disease 19-Associated Hospitalization Surveillance Network (COVID-NET) during the Omicron wave in the United States [7] but differs from earlier reports from South Africa that suggested Omicron was associated with a higher rate of incidental infection [10,11]. This may be due to a difference in the classification of indication for admission (COVID-19-related vs. incidental infection), or a difference in threshold for hospitalization or available hospital resources.

Moreover, there was no statistically significant difference in rates of hospitalization between vaccinated and unvaccinated children in our cohort. This is likely due to an overall low vaccination rate in our population; thus, our study may not have been powered to detect a difference in outcomes based on vaccination status. In contrast, a COVID-NET based study with a much larger cohort of children aged 5–11 years showed that COVID-19-associated hospitalization rates were approximately twice as high in unvaccinated compared to vaccinated children [20]. The vaccination rate in our patient population is low overall, potentially due to the nature of our data source (the IDR cannot identify if a child was vaccinated outside of New York State unless vaccination status is manually entered by a provider, for example). Another possibility is that because the COVID-19 vaccine was approved for children aged 5–11 years on 2 November 2021 in the U.S., many of our children may not have mounted an appropriate immune response because it had not been 2 weeks after the completed vaccine series prior to becoming infected with Omicron. While the majority of hospitalized patients were admitted for COVID-19-related indications, most of the admissions did not have severe COVID-19 disease, as most did not require respiratory support or ICU admission. Whether risk for severe COVID-19 disease in children is driven largely by vaccine status, pediatric-specific physiology and immune responses, or the specific variant itself, merits further exploration, especially as new variants continue to emerge.

Several limitations merit discussion. Our experience within our hospital system may not be generalizable. The threshold for hospitalization depends on clinician experience, clinician comfort level, and the availability of hospital resources and may differ among hospital systems based on geographic setting and community rates of COVID-19. Sequencing of SARS-CoV-2 PCR testing is ongoing in our cohort; therefore, we cannot be certain that COVID-19 cases were due to Omicron as opposed to another variant particularly in early cases. However, the rise in hospitalizations for COVID-19 positive patients in the second week of December is concurrent with Omicron dominance in NYC [1], increasing our confidence that we are primarily describing Omicron infections. Our methodology for attributing COVID-19-related vs. incidental hospitalizations is based on the prior literature and in-depth chart review; however, misclassification is possible. For example, although the prior literature demonstrates that COVID-19 exacerbates multiple chronic conditions [21,22], we cannot definitively conclude that admissions for exacerbation of chronic conditions were definitively due to COVID-19 infection.

In summary, most children hospitalized with COVID-19 during the Omicron wave from our ED were admitted for COVID-19-related indications as opposed to incidental COVID-19 infection. However, most hospitalized children did not require admission to the ICU or respiratory support. Children less than 5 years were impacted by higher rates of hospitalization and respiratory disease compared to older age groups. A minority of children with respiratory disease presented with croup. Our findings highlight the vulnerability of young children to COVID-19 hospitalizations and support the recent Advisory Committee on Immunization Practices recommendations for use of COVID-19 vaccine in children 6 months–4/5 years [23].

## Figures and Tables

**Table 1:** Demographic Characteristics of Patients Aged ≤18 years Presenting to Emergency Departments and Tested for SARS-CoV-2, 2 December 2021–23 January 2022.

	Overall (N = 2515)	COVID-19 Positive (N = 794)		
		Admitted (N = 81)	Not Admitted (N = 713)	*p*-Value ^b^
Age, median (IQR)	4.0 (2.0, 10.0)	4.0 (1.0, 11.0)	5.0 (2.0, 10.0)	0.3
**Agegroups** ^a^ N (%)				
0–4 years	1302 (51.8%)	49 (60%)	341 (47.8%)	
5–11 years	715 (28.4%)	15 (19%)	222 (31.1%)	
12–15 years	278 (11.1%)	6 (7%)	81 (11.4%)	
16–18 years	220 (8.7%)	11 (14%)	69 (9.7%)	
Sex, N (%)				0.4
Female	1174 (46.7%)	33 (41%)	322 (45.2%)	
Male	1341 (53.3%)	48 (59%)	391 (54.8%)	
**Race**, N (%)				0.12
American Indian or Alaska Native	20 (0.8%)	1 (1%)	7 (1.0%)	
Asian	105 (4.2%)	3 (4%)	31 (4.3%)	
Black Or African American	536 (21.3%)	14 (17%)	191 (26.8%)	
Native Hawaiian or Other Pacific Islander	3 (0.1%)	0 (0%)	1 (0.1%)	
White	940 (37.4%)	36 (44%)	222 (31.1%)	
Other	659 (26.2%)	16 (20%)	191 (26.8%)	
Unknown	252 (10.0%)	11 (14%)	70 (9.8%)	
**Ethnicity**, N (%)				0.066
Hispanic Or Latino or Spanish Origin	1144 (45.5%)	25 (31%)	316 (44.3%)	
Not Hispanic or Latino or Spanish Origin	1056 (42.0%)	44 (54%)	306 (42.9%)	
Unknown	315 (12.5%)	12 (15%)	91 (12.8%)	
**Medical complexity by Pediatric Medical** **ComplexityAlgorithm**, N (%)				<0.001
Complex chronic disease	796 (31.7%)	55 (68%)	173 (24.3%)	
Non-complex chronic disease	629 (25.0%)	11 (14%)	167 (23.4%)	
No chronic disease	1090 (43.3%)	15 (19%)	373 (52.3%)	
**Vaccinedoses** ^c^, n (%)	**n = 1213**	**n = 32**	**n = 372**	0.3
1	45 (3.7)	3 (9)	10 (2.7)	
2	287 (23.7)	6 (19)	74 (19.9)	
>2	23 (1.9)	0 (0)	5 (1.3)	
Unvaccinated ^d^	858 (70.7)	23 (72)	283 (76.1)	

IQR, interquartile range ^a^ Age groups divided according to vaccine eligibility. ^b^ Testing differences between admitted and not admitted COVID-19 positive patients. Wilcoxon rank sum test; Pearson’s Chi-squared test; Fisher’s exact test. ^c^ n includes only children eligible for vaccination (i.e., 5 years and older). ^d^ May include children who were vaccinated outside of New York State and have not yet had manual entry of their COVID-19 vaccine doses.

**Table 2:** Clinical Characteristics of Patients Aged ≤18 years Hospitalized for COVID-19-related Indication, 2 December 2021–23 January 2022.

	COVID-19-Related Admission (N = 58)
**Disposition**	
Any ICU stay, N (%)	11 (19)
Length of stay, median days (IQR)	3.2 (1.9, 4.7)
Discharged alive, N (%)	57 (98)
**Respiratory support**, N (%)	
No oxygen support	35 (60)
Nasal cannula or face mask	9 (16)
Non-invasive positive pressure ventilation	7 (12)
Invasive mechanical ventilation	6 (10)
**Treatment**, N (%)	
Remdesivir	19 (33)
Steroids, COVID-19 indication	12 (21)
Steroids, non-COVID-19 indication	13 (22)
Sotrovimab	1 (2)
**Primary diagnosis**, N (%)	
Croup	7 (12)
Other respiratory ^a^	21 (36)
Clinical monitoring ^b^	9 (16)
Exacerbation of underlying conditions ^c^	9 (16)
Neurologic ^d^	4 (7)
Other ^e^	8 (14)
**Viral co-infection**	
Viral co-infection ^f^, N (%)	12 (21)
Viral pathogen(s) detected (N)	
Adenovirus	0
Coronavirus 229E	0
Coronavirus HKU1	0
Coronavirus NL63	0
Coronavirus OC43	0
Influenza A	0
Influenza B	0
Parainfluenza 1	0
Parainfluenza 2	0
Parainfluenza 3	1
Parainfluenza 4	0
Human metapneumovirus	3
Respiratory syncytial virus	6
Rhinovirus/enterovirus	3

IQR, interquartile range. ^a^ Includes asthma exacerbations, pneumonia, bronchiolitis, and exacerbations of underlying respiratory conditions. ^b^ Clinical monitoring for medically complex patients. ^c^ Exacerbation of underlying chronic conditions without alternative explanation. ^d^ Includes bulging fontanelle and seizures. ^e^ Includes fever, dehydration, chest pain, or gastrointestinal symptoms. ^f^ At least one non-SARS-CoV-2 pathogen detected on Respiratory Pathogen Panel Test, Biofire RP2.1. Fifty-three out of fifty-eight patients had Respiratory Pathogen Panel Tests conducted. One patient had 2 viral co-infections. Remaining 5 patients had no viral co-infections detected with Cepheid Xpert Xpress CoV-2/Flu/RSV plus, which tests for SARS-CoV-2, influenza A and B, and RSV.

**Table 3:** Proportion of hospitalization in vaccine-eligible children (>5 years) who are not vaccinated compared to fully vaccinated children.

	Fully Vaccinated (2 or More Doses) N = 85	Not Fully Vaccinated (0 or 1 dose) N = 319	Risk Ratio (95% CI)
Hospitalized	6 (7%)	26 (8.2%)	1.2 (0.49, 2.7)
Not Hospitalized	79 (93%)	293 (91.8%)	

## Data Availability

The data presented in this study are available upon request from the corresponding author.
